# Supplementary material for: Association between age at first birth and risk of rheumatoid arthritis in women: Evidence from NHANES 2011–2020
Source: PLoS One. 2025 Jan 16;20(1):e0317443. doi: 10.1371/journal.pone.0317443 (PMC11737699; doi:10.1371/journal.pone.0317443)
Supplement: Attachment — Submitted filename: Response to Reviewers.docx [file pone.0317443.s002.docx]

[Aifeng Liu]

[First Teaching Hospital of Tianjin University of Traditional Chinese Medicine, Tianjin 300381, China]

[draifeng@163.com]

Editor-in-Chief,

PLOS ONE

Subject: Response to Reviewer Comments - Manuscript ID [PONE-D-24-53513]

Dear Editor-in-Chief,

We sincerely appreciate the valuable feedback provided by both the reviewers and the Editor for our manuscript titled " Association between age at first birth and risk of rheumatoid arthritis in women:evidence from NHANES 2011–2020" (Manuscript ID: PONE-D-24-53513). We have thoroughly reviewed and carefully considered all the comments received, and we have made comprehensive revisions to the manuscript, with text changes highlighted in red, to enhance its quality and clarity. We have meticulously reviewed the reference list to ensure its completeness and accuracy. Please find below our point-by-point response to the comments received:

****Reviewer 1:****

**[Minor comment 1]** • Please correct the abstract on the title page of the manuscript also.

**Response:** Thank you very much for your review. We have corrected the abstract on the title page of the manuscript.

****Reviewer 2:****

**[Comment 1]** •I found the revision sufficient.The authors have positively evaluated my suggested contributions in their articles. The changes made are satisfactory to me.

**Response:**Thank you for your feedback.

****Reviewer 3:****

**[Comment 1]** •1. Inconsistency in Claims about Previous Research
The introduction claims that no studies have specifically examined the relationship between Age at First Birth (AFB) and rheumatoid arthritis (RA) risk. However, the discussion section references several studies that indirectly or directly address this topic. This discrepancy undermines the novelty of the study and should be clarified.

**Response:** Thank you for your in-depth feedback on our study. We have made the necessary revisions to the introduction section of the manuscript.（Please refer to line 48 in the manuscript.）

**[Comment 2]** •2. Stylistic Deviations and Non-Academic Tone
The manuscript contains several instances of stylistic deviations from an academic tone, such as:
a) The phrase, "Policymakers must focus more on women in these AFB age ranges in screening RA and making preventive measures," employs emotionally charged language ("must") and shifts the tone from scientific to political. A more neutral phrasing is recommended.
b) A subjective critique of Zhu et al.'s study, emphasizing insufficient sample size without detailed justification.
c) Statements regarding the increased risk due to late childbirth ("longer period to accumulate exposures to environmental and lifestyle factors") presented as factual without sufficient evidence.
d) These examples are not exhaustive; the authors are encouraged to thoroughly review the manuscript for similar stylistic deviations to enhance its academic rigor.

**Response:** Thank you for your valuable feedback on the article.

a) We have made the necessary revisions to the conclusion section of the manuscript.

（Please refer to line 34 in the manuscript.）

b) We have provided an explanation for the study conducted by Zhu et al.

（Please refer to line 143 in the manuscript.）

c)We have supplemented the discussion section of the article with relevant references to support our conclusions.

References：

[30]Winkler-Dworak M, Pohl M, Beaujouan E. Scenarios of Delayed First Births and Associated Cohort Fertility Levels. Demography. 2024 Jun;61: 687–710. doi:10.1215/00703370-11315685.

[31]Ritonja JA, Madathil S, Nicolau B, L’Espérance K, Ho V, Abrahamowicz M, et al. Body fatness across the adult life course and ovarian cancer risk. Eur J Epidemiol. 2024 Oct;39: 1139–1149. doi:10.1007/s10654-024-01161-1.

d)Thank you for your review. We have conducted a thorough examination of the entire manuscript to ensure its academic rigor.

**[Comment 3]** •3. Lack of Control for Multiple Comparisons and Type I Errors
The manuscript reports multiple statistically significant findings (P < 0.05) across various subgroups and categories but does not address whether adjustments for multiple comparisons were applied. This raises concerns about inflated Type I error rates (false positives). The absence of correction methods like Bonferroni or Holm adjustments undermines the validity of some conclusions, particularly given the extensive number of statistical tests performed. The authors should clarify whether such adjustments were applied or acknowledge this as a limitation in the discussion.

**Response:** We appreciate the reviewer’s rigorous suggestions regarding the statistical analysis in this manuscript. This feedback is highly valuable for improving both the current study and our future research. It is true that Bonferroni correction or other multiple comparison adjustments were not applied when analyzing variables with multiple subgroups, which represents a limitation of this study. However, most of the p-values for the variables included in our analyses were less than 0.001, indicating statistically significant differences with a very low probability of Type I error. Therefore, even without multiple comparison adjustments, the potential bias introduced in this study is minimal, and the overall impact on the conclusions is likely negligible.

Additionally, we note that other studies utilizing the NHANES database have similarly not universally applied Bonferroni correction for subgroup comparisons. Nevertheless, we recognize the importance of addressing multiple comparisons and will prioritize this aspect in future studies to further enhance the rigor and reliability of our research.

References：

[1] Paulose-Ram R, Graber JE, Woodwell D, Ahluwalia N. The National Health and Nutrition Examination Survey (NHANES), 2021-2022: Adapting Data Collection in a COVID-19 Environment. Am J Public Health. 2021 Dec;111(12):2149-2156. doi: 10.2105/AJPH.2021.306517. PMID: 34878854; PMCID: PMC8667826.
[2] Liu Z, Kuo PL, Horvath S, Crimmins E, Ferrucci L, Levine M. A new aging measure captures morbidity and mortality risk across diverse subpopulations from NHANES IV: A cohort study. PLoS Med. 2018 Dec 31;15(12):e1002718. doi: 10.1371/journal.pmed.1002760. PMID: 30596641; PMCID: PMC6312200.

We are confident that these revisions have substantially enhanced the manuscript and brought it in line with the standards set by PLOS ONE.

Enclosed with this response letter, please find the updated manuscript, including all necessary changes and tracked revisions. We believe that these modifications effectively address the feedback received.

At the same time, we would like to thank the reviewers and the editor for their constructive feedback and guidance, which have greatly contributed to the refinement of our work.

We appreciate the opportunity to revise and resubmit our manuscript, and we are hopeful for a positive outcome. We remain committed to addressing any further suggestions or recommendations from the reviewers and the Editor. Thank you for your consideration.

Sincerely,

Aifeng Liu
